# Supplementary material for: Analysis of Perception, Reasons, and Motivations for COVID-19 Vaccination in People with Diabetes across Sub-Saharan Africa: A Mixed-Method Approach
Source: Int J Environ Res Public Health. 2022 Jun 27;19(13):7875. doi: 10.3390/ijerph19137875 (PMC9266073; doi:10.3390/ijerph19137875)

Supplementary File

Supplementary Table S1: Participants comments on reasons for their unwillingness to accept the COVID-19 vaccines (n=19).

Reasons (Themes)	Statements
Mistrust for the country where the vaccine was produced	Why is the European countries that are the most affected refusing to take the vaccine, but are interested in making the vaccine and sending to Africa. Do you think we can trust them?<br>Why is the Western country much interested in Africa meanwhile, we are less affected than the countries producing the vaccines. Europeans should take the vaccine first not forcing it on Africa
Mistrust for the Pharmaceutical Company	Manufacturing companies' refusal to take responsibility for any adverse effects arising from taking the vaccines<br>The indemnity given to manufactures is curious and suspicious
Mistrust in the medical process for developing the vaccine	Not enough scientific research results<br>The vaccines have been developed so quickly. I don't trust the research done about it.<br>Not enough study on the long-term effect of the vaccines<br>Not enough scientific data on clinical trials; Not sufficient time elapsed for observations of side-effects
Mistrust of the health system in my country	My country is making money with covid 19. No trace of it kind here
Personal beliefs/past historical experiences with vaccines	Vaccines have been used against black people for far too long - Kenya infertility, Tuskegee, etc. This vaccine is as questionable and its benefits for politicians far outweigh its care to manage this self-limiting bug.<br>personal intuition, personal decision, personal choice, personal beliefs, personal conviction that the vaccine is not necessary in Africa especially for young people who are not at risk
Concerned about safety of the COVID-19 vaccine	Real benefits of the vaccines not spelt out. Vaccines shrouded with uncertainties; Side effects have a history of thrombosis<br>Risk to my health as I have SLE with a severely compromised immune system; Pregnant; there have also been new developments about how those vaccinated have been re-infected with the virus.
Advice from religious leaders	Personal belief in my Lord Jesus Christ
Conspiracy theories	The conspiracy theories that are circulating regarding COVID-19 vaccine have discouraged me from taking the vaccine<br>It could be a birth control procedure to reduce world population<br>The vaccine is meant to reduce world population especially Africans<br>I am protected from complications of the Covid virus<br>If this vaccine is not curative and not preventive, what does it do?

Supplementary Figure S1: Percentage breakdown of internet sources used by the participants (n=64) to retrieve COVID-19 vaccine related information during the pandemic. Totals exceed 100% due to multiple responses.

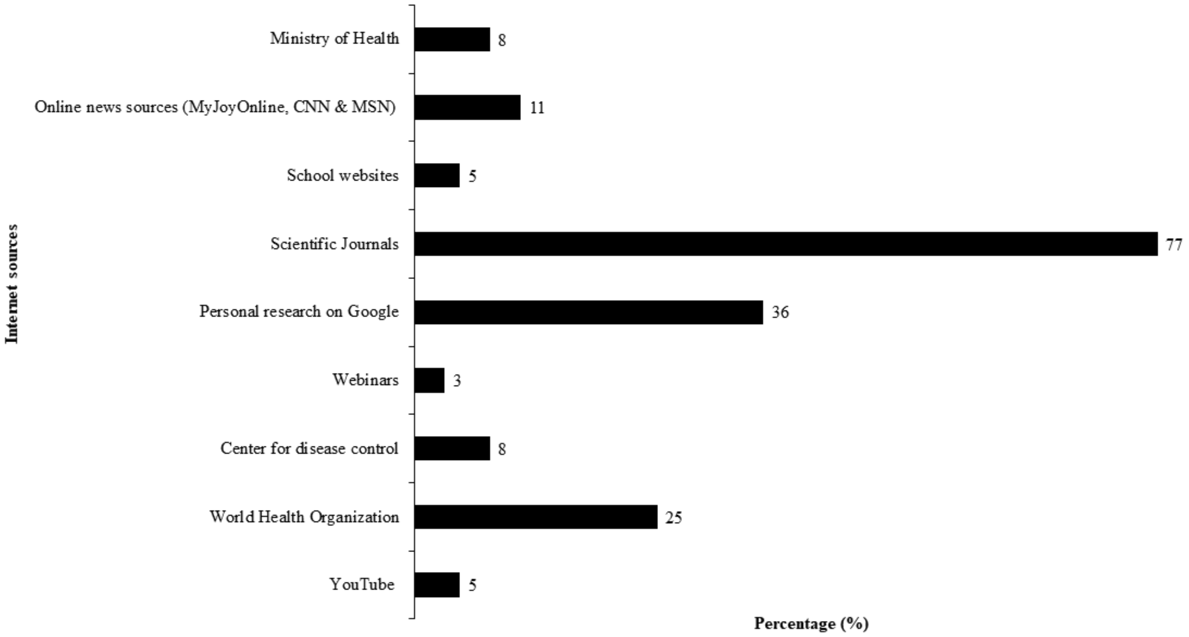

Supplement: Supplementary file 1 [file ijerph-19-07875-s001.zip › ijerph-1795043-supplementary.pdf]
